# Pharmacologic Disruption: How Emerging Weight Loss Therapies Are Challenging Bariatric Surgery Guidelines

**DOI:** 10.3390/medicina61071292

**Published:** 2025-07-18

**Authors:** Safi G. Alqatari, Abrar J. Alwaheed, Manal A. Hasan, Reem J. Al Argan, Marj M. Alabdullah, Mohammed D. Al Shubbar

**Affiliations:** Department of Internal Medicine, College of Medicine, Imam Abdulrahman Bin Faisal University, King Fahd University Hospital, Dammam 31441, Eastern Province, Saudi Arabia; sagqatari@iau.edu.sa (S.G.A.); ajwaheed@iau.edu.sa (A.J.A.); mahasan@iau.edu.sa (M.A.H.); rjalarqan@iau.edu.sa (R.J.A.A.); marjalabdullah@gmail.com (M.M.A.)

**Keywords:** anti-obesity agents, bariatric surgery, obesity, weight loss medications, glucagon-like peptide 1

## Abstract

Obesity is a chronic, relapsing disease with multifactorial origins and significant global health implications. Historically, bariatric surgery has been the most effective intervention for achieving sustained weight loss and metabolic improvement, especially in individuals with moderate to severe obesity. However, the therapeutic landscape is rapidly evolving. Recent advances in pharmacotherapy—including GLP-1 receptor agonists, dual and triple incretin agonists, and amylin-based combination therapies—have demonstrated unprecedented efficacy, with some agents inducing 15–25% weight loss, approaching outcomes once exclusive to surgical intervention. These developments challenge the continued applicability of existing bariatric surgery criteria, which were established in an era of limited medical alternatives. In this narrative review, we examine the evolution of surgical eligibility thresholds and critically assess the potential role of novel pharmacotherapies in redefining treatment algorithms. By comparing the efficacy, safety, metabolic benefits, and cost-effectiveness of surgery versus next-generation drugs, we explore whether a more stepwise, pharmacotherapy-first approach may now be justified, particularly in patients with BMI 30–40 kg/m^2^. We also discuss future directions in obesity management, including personalized treatment strategies, perioperative drug use, and the integration of pharmacologic agents into long-term care pathways. As the field advances, a paradigm shift toward individualized, minimally invasive interventions appears inevitable—necessitating a timely re-evaluation of current bariatric surgery guidelines to reflect the expanding potential of medical therapy.

## 1. Introduction

Obesity is a growing global health problem and is now considered a chronic epidemic. Excessive adiposity significantly increases the risk of various chronic diseases, including type 2 diabetes mellitus, cardiovascular disease, non-alcoholic fatty liver disease, and certain cancers—ultimately contributing to reduced quality of life and rising healthcare costs [1]. Its complex etiology involves a combination of genetic predisposition, environmental exposures, sedentary lifestyles, and the widespread consumption of calorie-dense, nutrient-poor diets, all of which have fueled the persistence and severity of this global crisis [2].

The global burden of obesity continues to escalate at an alarming rate. As of 2022, approximately 1 in 8 individuals worldwide were living with obesity, with adult obesity rates more than doubling and adolescent obesity quadrupling since 1990. An estimated 2.5 billion adults were overweight in 2022, including 890 million classified as obese—accounting for 43% and 16% of the adult population, respectively. In 2021 alone, elevated body mass index (BMI) contributed to approximately 3.7 million deaths due to noncommunicable diseases, including cardiovascular disease, diabetes, cancer, neurological disorders, and chronic respiratory and digestive conditions [3]. Future projections indicate that over 51% of the global population will be overweight or obese by 2035 [4].

Historically, obesity has been difficult to manage. Lifestyle changes alone often result in modest and short-lived weight loss, while early pharmacologic treatments were limited by low efficacy or significant safety concerns—such as psychiatric side effects and poor tolerability seen with sympathomimetics, endocannabinoid blockers, and lipase inhibitors [5]. For decades, bariatric surgery has been recognized as the most effective and lasting treatment for obesity, consistently achieving substantial weight loss and remission of obesity-related comorbidities in cases where medical therapy had proven insufficient [6]. However, although bariatric surgery is an effective intervention for managing severe obesity, its global utilization remains strikingly limited. According to the World Health Organization, approximately 43% of adults worldwide were classified as overweight in 2022, with 16% meeting criteria for obesity [3]. Despite this alarming prevalence, only 468,609 bariatric procedures were performed globally in 2013, accounting for a mere 0.01% of the world’s population [7].

Saudi Arabia exemplifies this discrepancy. With an adult obesity prevalence of 35.4% [8], it ranks among the top countries in terms of bariatric surgery volume, performing 13,194 procedures in 2013. However, this figure represents just 0.0458% of the national population—an unexpectedly low rate given the country’s substantial obesity burden. Even lower rates are seen in resource-constrained countries, where access to surgical interventions is further limited [7].

This profound mismatch between the global burden of obesity and the limited application of metabolic surgery underscores a critical gap in the treatment landscape, revealing the urgent need for strategies to expand equitable access to effective obesity interventions.

Recent advancements in pharmacotherapy—particularly the development of hormone-based agents that modulate appetite and metabolism—have significantly improved weight loss outcomes. Glucagon-like peptide-1 (GLP-1) receptor agonists, along with newer multi-receptor agonists, have shown marked efficacy in clinical trials [9], suggesting a potential shift in the therapeutic landscape previously dominated by surgical interventions. This transformative shift in the therapeutic landscape necessitates a critical reassessment of current treatment algorithms and the criteria for surgical referral.

In this context, our review aims to critically examine whether current bariatric surgery guidelines remain appropriate in the era of increasingly potent pharmacologic therapies. We begin by outlining the evolution and rationale behind existing surgical criteria, followed by an in-depth analysis of emerging anti-obesity medications, categorized by their mechanisms of action and supported by recent clinical trial data. We then compare these pharmacologic approaches to surgical interventions across key domains—including efficacy, safety, metabolic outcomes, and cost-effectiveness. Special emphasis is placed on how these advancements may influence the development and refinement of treatment algorithms, including the potential redefinition of BMI thresholds and a shift toward more individualized, stepwise care. Ultimately, this review explores how the expanding therapeutic landscape may reshape clinical decision-making and inform future guideline development.

## 2. Search Methodology

This narrative review was informed by a structured literature search conducted across four major databases: PubMed, EMBASE, Web of Science (WOS), and Google Scholar, covering studies published between January 2010 and May 2025. The search was performed between January and May 2025 using a combination of MeSH terms and free-text keywords, including but not limited to the following: obesity, overweight, BMI, bariatric surgery, metabolic surgery, gastric bypass, sleeve gastrectomy, GLP-1 receptor agonists, semaglutide, liraglutide, tirzepatide, mazdutide, retatrutide, survodutide, incretin-based therapy, dual and triple agonists, amylin analogues, CagriSema, bimagrumab, myostatin inhibitors, FGF21 analogues, PYY analogues, ARD-101, TAS2R agonists, anti-obesity drugs, pharmacotherapy, guideline revision, BMI threshold, surgical eligibility, comparative efficacy, and cost-effectiveness. We prioritized peer-reviewed original research articles, randomized controlled trials (RCTs), meta-analyses, systematic reviews, and official clinical guidelines. Articles not published in English, as well as preprints and non-peer-reviewed materials, were excluded unless they offered critical preliminary data. Reference lists of included studies were also reviewed to identify additional relevant sources.

## 3. Current Bariatric Surgery Guidelines and Rationale of the 2022 ASMBS/IFSO Change

Contemporary bariatric surgery guidelines have progressively broadened eligibility criteria, largely in response to the historical scarcity of effective non-surgical treatments. The original 1991 NIH consensus limited surgical intervention to individuals with a body mass index (BMI) ≥ 40 kg/m^2^, or ≥35 kg/m^2^ in the presence of at least one serious obesity-related comorbidity [10]. However, accumulating evidence over the past decades has demonstrated the substantial benefits of metabolic surgery, even in individuals with less severe obesity. Reflecting this paradigm shift, the 2022 joint guidelines from the American Society for Metabolic and Bariatric Surgery (ASMBS) and the International Federation for the Surgery of Obesity and Metabolic Disorders (IFSO) now recommend metabolic surgery for all patients with a BMI ≥ 35 kg/m^2^, irrespective of the presence, absence, or severity of comorbid conditions. Furthermore, surgery should be considered in patients with a BMI of 30–34.9 kg/m^2^ who have metabolic diseases such as type 2 diabetes, and may also be appropriate for select patients in this BMI range without metabolic disease if they fail to achieve meaningful or sustained weight loss through non-surgical approaches. Notably, for Asian populations, BMI thresholds are approximately 2.5 kg/m^2^ lower due to higher metabolic risk at lower BMI values. These revisions are grounded in data from randomized controlled trials and large cohort studies demonstrating that metabolic surgery leads to significant and durable weight loss, remission of type 2 diabetes, and reductions in cardiovascular risk, even among patients with class I obesity [11].

The rationale for lowering BMI thresholds for bariatric surgery stemmed from the historical inadequacy of lifestyle interventions and earlier pharmacologic agents in achieving sustained weight loss among lower-BMI patients with metabolic disease [11]. In this therapeutic void, surgery emerged as a superior option. Clinical trials subsequently confirmed that procedures such as Roux-en-Y gastric bypass and sleeve gastrectomy resulted in significantly greater weight loss and glycemic control compared to medical therapy alone in individuals with type 2 diabetes and a BMI < 35 kg/m^2^ [11]. These findings prompted the inclusion of metabolic surgery as a recommended intervention for patients with moderate obesity and associated comorbidities, or for those who failed to respond adequately to non-surgical approaches. For individuals with a BMI ≥ 35 kg/m^2^, surgery is now strongly recommended, supported by robust evidence demonstrating long-term safety, improvements in quality of life, and reductions in mortality. Until very recently, bariatric surgery remained the most effective evidence-based intervention for obesity across all BMI categories [11].

## 4. Paradigm Shift Toward Pharmacotherapy

While the evolution of bariatric surgery guidelines—from the original NIH 1991 consensus to the broader 2022 ASMBS/IFSO recommendations—has been well-justified by a growing body of evidence [11], it is important to recognize that several factors are prompting a concurrent shift in focus toward pharmacotherapy. This shift is not intended to diminish the value of metabolic surgery, but rather to support a more integrated, accessible, and individualized approach to obesity treatment in which pharmacologic and surgical modalities work synergistically rather than in isolation.

A key force behind this transition is the limited global accessibility of bariatric surgery. Despite its established efficacy, the procedure remains dramatically underutilized. In 2013, only 468,609 bariatric operations were performed globally—representing a mere 0.01% of the world’s population [7]. This profound mismatch illustrates a massive treatment gap. Even in countries with high surgical volumes and high resources, such as Saudi Arabia—with an obesity prevalence of 35.4% [8]—only 0.0458% of the population underwent bariatric surgery in 2013 [7]. Rates are even lower in resource-limited settings, where healthcare infrastructure, surgical capacity, and economic constraints further restrict access.

Second, the cost of bariatric surgery and non-coverage by insurance companies remains prohibitive in many healthcare systems. While bariatric surgery has been shown to be cost-effective in the long term [12] and is sometimes covered under public and private insurance plans in certain regions, coverage remains inconsistent and frequently insufficient. In the United States, for example, financial hardship and insurance non-coverage were among the most commonly self-reported reasons for not pursuing surgery, even among patients who were referred and deemed eligible by a bariatric surgeon. In a single-institution study conducted in a U.S. metropolitan area, only 45% of eligible patients progressed to surgery despite being evaluated by a bariatric surgeon [13]. Other frequently patient-reported barriers include fear of complications and wait time between evaluation and surgery [14].

Third, in light of the significant barriers that limit access to bariatric surgery, including cost, insurance hurdles, and geographic disparities, pharmacotherapy emerges as an increasingly viable and scalable alternative, with several novel agents already demonstrating remarkable efficacy. GLP-1 receptor agonists such as semaglutide and dual agonists like tirzepatide have shown average weight reductions of up to 20% in clinical trials [15]—results that approach those seen with surgical interventions. Importantly, many of these agents also confer clinically meaningful benefits beyond weight loss, including reduction in cardiovascular events, and slowing the progression of chronic kidney disease [16], among other benefits that will be discussed in the next section.

In addition to their efficacy, these newer agents are generally well-tolerated and less invasive, offering a treatment modality that is more acceptable to a wider range of patients, especially those hesitant to undergo surgery. For both patients and clinicians, this presents a compelling alternative that may better align with individual preferences, medical comorbidities, or treatment goals.

These developments raise fundamental questions about the future direction of obesity treatment guidelines. Should we re-evaluate the current BMI-based cutoffs for bariatric surgery eligibility? Is it still justified to proceed with metabolic surgery at the expanded thresholds introduced in recent guidelines, or should we consider reverting to more conservative criteria in light of the potency of emerging pharmacotherapies? Alternatively, would the development of integrated, hybrid treatment guidelines—that combine pharmacologic and surgical strategies in a more coordinated fashion—provide a better framework for optimizing outcomes? A new model may be needed, one that goes beyond BMI alone and incorporates patient preferences, comorbidity burden, obesity phenotypes, and treatment response into clinical decision-making. In this evolving landscape, treatment algorithms should be patient-centred, comorbidity-driven, and flexible, enabling clinicians to tailor interventions across a continuum of care rather than forcing binary choices between medical or surgical approaches. Such a shift would not only reflect the complexity of obesity as a chronic, multifactorial disease but also ensure that both medical and surgical therapies are used strategically—and synergistically—to maximize patient benefit.

This paradigm shift ultimately supports a more comprehensive and layered approach, in which pharmacotherapy is not seen as a replacement for surgery, but as a critical tool to improve access, personalize care, and enhance long-term outcomes—whether used independently or as part of a stepped-care model leading to or complementing surgery.

## 5. Current and Emerging Pharmacotherapies for Obesity: Mechanisms and Clinical Data

A new generation of pharmacologic agents is reshaping the landscape of obesity management. Many of these compounds, initially developed for the treatment of type 2 diabetes, act on hormonal pathways that modulate appetite, satiety, and energy expenditure. In the following sections, we provide a structured overview of the most promising therapies, categorized by their mechanisms of action. For each agent, we summarize its pharmacological target, stage of clinical development or approval, efficacy as demonstrated in peer-reviewed clinical trials, and safety profile. This review emphasizes evidence from high-quality studies to inform current and emerging therapeutic strategies for obesity.

### 5.1. GLP-1 Receptor Agonists (Monotherapy)

Glucagon-like peptide-1 (GLP-1) receptor agonists enhance glucose-dependent insulin secretion and activate central satiety pathways, thereby reducing appetite and caloric intake. Additionally, they delay gastric emptying, contributing further to weight loss [17,18]. Historically, the therapeutic landscape was limited to injectable peptide-based GLP-1 analogues, such as liraglutide and semaglutide. However, recent advancements have introduced oral non-peptide GLP-1 receptor agonists and next-generation injectable formulations, broadening both the pharmacologic toolkit and patient accessibility.

#### 5.1.1. Semaglutide

Semaglutide, a glucagon-like peptide-1 (GLP-1) receptor agonist, has demonstrated substantial and sustained efficacy in promoting weight loss among adults with overweight or obesity. Administered at a dose of 2.4 mg once weekly in conjunction with lifestyle modifications, semaglutide achieved a mean weight reduction of 14.9%, with more than 86% of participants attaining a weight loss of at least 5%, and over half achieving a reduction of 15% or greater. The most commonly reported adverse events were nausea and diarrhea, which were typically mild-to-moderate in severity and transient in nature [19].

Subsequent clinical investigations have expanded the understanding of semaglutide’s therapeutic potential beyond weight loss. Notably, the FLOW trial revealed that semaglutide significantly reduced the risk of major kidney disease events by 24%, cardiovascular mortality by 29%, and all-cause mortality by 20%. In addition to these outcomes, semaglutide was associated with a slower annual decline in estimated glomerular filtration rate (eGFR), a reduction in major adverse cardiovascular events, improved glycemic control, decreased albuminuria, and an overall favourable safety profile [16]. These cardiorenal benefits are further supported by additional large-scale cardiovascular outcome trials. The PIONEER-6, SUSTAIN-6, and SOUL trials have all confirmed the cardiovascular safety of semaglutide, with consistent reductions in major adverse cardiovascular events across diverse patient populations [20,21,22]. Beyond its cardiometabolic impact, semaglutide has also demonstrated potential in musculoskeletal health. In patients with knee osteoarthritis, it was shown to significantly alleviate pain and enhance functional status, indicating possible utility in managing obesity-related osteoarthritic symptoms [23].

Furthermore, emerging real-world evidence supports the role of semaglutide in augmenting weight loss even in individuals with prior metabolic bariatric surgery. A recent study involving approximately 1000 patients demonstrated that those with a history of bariatric surgery experienced significantly greater weight loss with semaglutide compared to those without prior surgery (13.6% vs. 10.1%) [24]. These findings highlight the potential additive or synergistic effects of integrating pharmacotherapy with surgical interventions, thereby reinforcing the importance of a multimodal and individualized approach to obesity management when clinically appropriate.

#### 5.1.2. Orforglipron (LY3502970)

Orforglipron is the first oral, non-peptide GLP-1 receptor agonist to advance to clinical trials. In a Phase 2 study, once-daily orforglipron demonstrated dose-dependent weight loss comparable to that of injectable GLP-1 analogues. By week 36, mean body weight reduction ranged from −9.4% to −14.7% across the 12–45 mg dose groups, compared to −2.3% in the placebo arm. Notably, nearly 75% of participants receiving higher doses achieved ≥10% weight loss within 36 weeks. Efficacy was already apparent by week 26, with weight loss ranging from −8.6% to −12.6% in the orforglipron groups versus –2.0% for placebo. The safety profile was consistent with the known effects of GLP-1 receptor agonists; the most frequently reported adverse events were gastrointestinal—primarily nausea and diarrhea—which were generally mild to moderate in severity and occurred predominantly during the dose-escalation period. Importantly, no new safety signals, such as pancreatitis or serious adverse events, were identified. As a once-daily oral agent, orforglipron offers a promising alternative to injectable therapies and may significantly improve accessibility and adherence. Given the robust weight reduction observed—approaching 15%—Phase 3 trials are currently underway to further evaluate its efficacy and long-term safety [25].

#### 5.1.3. Danuglipron (PF-06882961)

Danuglipron is an orally administered small-molecule GLP-1 receptor agonist developed by Pfizer. Early-phase clinical trials demonstrated promising glycemic and weight-lowering effects; however, tolerability has emerged as a significant limitation. In a 12-week Phase 2a trial, participants titrated to 200 mg twice daily experienced notably higher rates of gastrointestinal adverse events—particularly nausea and vomiting—resulting in substantially increased dropout rates compared to placebo. Despite these challenges, danuglipron produced meaningful metabolic benefits: among individuals with type 2 diabetes, hemoglobin A1c levels decreased by approximately 1.0% to 1.6%, and body weight was reduced by 1.9 to 5.4 kg (up to ~5% of body weight) over the 12-week period, while placebo-treated participants exhibited minimal change [26]. A parallel cohort of obese, nondiabetic individuals receiving the same dose also experienced weight loss, with unpublished Phase 2b data reportedly showing reductions of approximately 8% to 13% at 32 weeks [27]. However, discontinuation rates remained high, ranging from 27% to 73% in the treatment arms versus approximately 17% for placebo, largely due to gastrointestinal side effects [26]. In response, the developer has shifted focus toward an extended-release formulation aimed at further optimizing pharmacokinetic properties [28]. If gastrointestinal adverse events can be mitigated, danuglipron may represent a viable oral GLP-1 agonist. However, current data suggest it is less well-tolerated than orforglipron [26].

#### 5.1.4. Ecnoglutide (XW003)

Ecnoglutide is a long-acting, once-weekly GLP-1 receptor agonist under development by Sciwind Biosciences. Phase 2 clinical trials conducted in China have primarily targeted patients with type 2 diabetes and obesity. In a 20-week randomized study involving individuals with type 2 diabetes, weekly administration of ecnoglutide at a dose of 1.2 mg led to a substantial reduction in HbA1c levels, with a mean decrease of 2.39% compared to −0.55% in the placebo group [29]. While glycemic control was pronounced, weight loss in this cohort was relatively modest: 33% of patients receiving ecnoglutide achieved ≥5% body weight reduction, compared to 3% in the placebo group. These findings highlight ecnoglutide’s strong glucose-lowering efficacy, with moderate weight-reducing effects in a diabetic population [29].

#### 5.1.5. Mazdutide (IBI362)

Mazdutide is a dual GLP-1 and glucose-dependent insulinotropic polypeptide (GIP) receptor agonist, often classified alongside GLP-1 receptor agonists due to its overlapping mechanisms. Developed by Innovent Biologics, mazdutide has demonstrated substantial weight-reducing effects in clinical trials conducted in China. In a 24-week Phase 2 study, mazdutide induced dose-dependent weight loss: −6.7% at 3 mg, −10.4% at 4.5 mg, and –11.3% at 6 mg administered once weekly, compared to a 1.0% weight gain in the placebo group [30]. A separate cohort receiving a higher 9 mg dose, specifically in patients with obesity and non-alcoholic fatty liver disease, achieved even greater reductions, with a mean weight loss of 13.3% and 31.7% of participants attaining ≥15% weight loss [31]. Mazdutide was generally well-tolerated, with gastrointestinal adverse events being the most common, consistent with the safety profile of other incretin-based therapies [30]. These findings suggest that mazdutide’s efficacy in mid-phase trials is comparable to that of high-dose semaglutide and tirzepatide. A Phase 3 clinical programme is currently underway in China. Mazdutide exemplifies the therapeutic potential of dual incretin receptor agonism, with co-activation of GLP-1 and GIP pathways modestly enhancing weight loss beyond that achieved with GLP-1 monotherapy.

### 5.2. Dual and Triple Incretin Receptor Agonists

Multi-agonist therapies targeting combinations of gut hormone receptors—such as GLP-1, glucose-dependent insulinotropic polypeptide (GIP), and glucagon receptors—represent a novel approach to enhancing metabolic outcomes in obesity treatment. By concurrently engaging these pathways, these agents aim to achieve additive or synergistic effects on weight reduction and metabolic regulation. GLP-1 and GIP receptor agonism primarily suppress appetite and enhance satiety, while glucagon receptor activation promotes increased energy expenditure and fat oxidation. However, glucagon-induced hyperglycemia remains a potential risk when not adequately counterbalanced [32]. The following section outlines key multi-receptor agonists currently in development, focusing on their mechanisms of action, clinical efficacy, and therapeutic potential.

#### 5.2.1. Tirzapatide

Tirzepatide represents a significant advancement in the pharmacologic management of obesity, distinguishing itself as one of the first anti-obesity agents to achieve weight loss outcomes approaching those typically observed with bariatric surgery. In the phase 3 SURMOUNT-1 trial, adults with obesity or overweight and at least one weight-related comorbidity (excluding diabetes) were randomized to receive once-weekly subcutaneous tirzepatide at doses of 5 mg, 10 mg, or 15 mg over a 72-week period. The intervention resulted in substantial and dose-dependent reductions in body weight, with mean losses of 15.0%, 19.5%, and 20.9%, respectively, compared to 3.1% with placebo. Notably, 50–57% of individuals in the 10 mg and 15 mg groups achieved a weight reduction of 20% or more—an outcome previously attainable primarily through metabolic surgery [33].

Beyond its profound effects on weight, tirzepatide has demonstrated therapeutic potential across multiple obesity-related comorbidities. In the SURMOUNT-OSA trials, conducted in adults with obesity and moderate-to-severe obstructive sleep apnea (OSA), tirzepatide significantly reduced the apnea–hypopnea index (AHI) relative to placebo. These improvements were accompanied by reductions in body weight, hypoxic burden, systolic blood pressure, and improvements in patient-reported sleep quality [34].

Additionally, tirzepatide has shown promise in the treatment of metabolic dysfunction-associated steatohepatitis (MASH). In the phase 2 SYNERGY-NASH trial, patients with biopsy-confirmed MASH and stage F2 or F3 fibrosis who received tirzepatide once weekly demonstrated significantly higher rates of MASH resolution without fibrosis progression. At 52 weeks, up to 62% of participants in the 15 mg group achieved histologic resolution of MASH, compared to only 10% in the placebo arm [35]. These findings collectively underscore the growing body of evidence supporting the pleiotropic nature of GLP-1 receptor agonism, with emerging data demonstrating therapeutic benefits that extend well beyond glycemic control and weight loss.

#### 5.2.2. Retatrutide (LY3437943)

Retatrutide is a first-in-class triple agonist targeting the GLP-1, GIP, and glucagon receptors—an approach often referred to as “triple G” agonism. In a landmark Phase 2 clinical trial, once-weekly retatrutide demonstrated unprecedented efficacy in weight reduction. At 48 weeks, patients receiving the 12 mg dose achieved a mean weight loss of 24.2% from baseline, compared to approximately 2% in the placebo group. Notably, 83% of individuals in the 12 mg cohort lost ≥ 15% of their initial body weight. Even lower doses, such as 4 mg and 8 mg, yielded impressive reductions of approximately 17% to 23%. Importantly, weight loss continued progressively throughout the 48-week treatment period, with no clear plateau reached—suggesting the potential for even greater efficacy with longer treatment duration [36]. This observation aligns with prior experience from other highly efficacious agents, such as tirzepatide, where extended use resulted in further weight reduction in Phase 3 trials [33]. The magnitude of weight loss achieved with retatrutide rivals outcomes typically associated with bariatric surgery. Beyond its effects on body weight, retatrutide also conferred significant metabolic benefits, particularly in improving glycemic control and other cardiometabolic risk markers. While mild, transient increases in heart rate were observed—a known class effect associated with GLP-1 and glucagon receptor activation—no serious safety concerns emerged in the Phase 2 study. Retatrutide’s ability to induce ~24% weight loss within one year represents a potential watershed moment in obesity pharmacotherapy, redefining expectations for non-surgical interventions [9,36].

#### 5.2.3. Survodutide (BI 456906)

Survodutide is a dual GLP-1 and glucagon receptor agonist developed by Boehringer Ingelheim, designed to leverage the complementary effects of appetite suppression and increased energy expenditure. In a 46-week Phase 2 trial involving individuals with overweight or obesity, once-weekly administration of survodutide resulted in substantial, dose-dependent weight loss. At the highest tested dose (4.8 mg), participants achieved a mean body weight reduction of 14.9% from baseline, compared to 2.8% in the placebo group—a placebo-adjusted difference of approximately 12.1%. More than half of the patients in the 4.8 mg group experienced weight loss of 15% or more. Clinically meaningful improvements were also observed in waist circumference and blood pressure. Notably, weight reduction of ~12–13% was already seen at intermediate doses (2.4 mg and 3.6 mg), suggesting diminishing marginal benefit at higher doses, which were associated with increased adverse effects. Gastrointestinal side effects were common, with 75% of survodutide-treated participants (all doses pooled) reporting gastrointestinal disorders—primarily nausea—compared to 42% in the placebo group. Overall, survodutide demonstrated promising efficacy, achieving approximately 15% weight loss over 46 weeks, and is currently being evaluated in Phase 3 trials for both obesity and non-alcoholic steatohepatitis (NASH) [37].

#### 5.2.4. VK2735

VK2735 is a dual GLP-1 and GIP receptor agonist developed by Viking Therapeutics, administered as a once-weekly subcutaneous injection. Although still in early clinical development, VK2735 has demonstrated impressive efficacy in initial trials. In the 13-week Phase 2 VENTURE study, VK2735 achieved up to 14.7% weight loss from baseline, with 88% of participants losing ≥10% of their body weight, compared to only 4% in the placebo group. The agent was generally well tolerated, with a safety profile consistent with other incretin-based therapies—predominantly mild gastrointestinal adverse events. Notably, no serious adverse events were reported. These early findings suggest that VK2735 may offer efficacy comparable to established combination therapies such as tirzepatide, with the added potential for both injectable and oral formulations under development. The substantial ~15% weight reduction observed within just three months highlights VK2735’s potency, though gastrointestinal tolerability will remain a key consideration as the programme advances into larger, longer-term trials [38].

#### 5.2.5. Maridebart Cafraglutide (AMG 133, “MariTide”)

AMG 133, also known as “MariTide,” is a novel bispecific agent that combines GLP-1 receptor agonism with GIP receptor antagonism—a unique strategy aimed at enhancing weight loss by simultaneously stimulating and inhibiting distinct components of the incretin axis. The molecule comprises a monoclonal antibody targeting the GIP receptor conjugated to GLP-1 analogue peptides. In a Phase 1 clinical trial involving individuals with obesity, monthly administration of AMG 133 led to dose-dependent weight loss without major safety concerns [39].

More compelling results were reported from a Phase 2 study recently presented by Amgen: after 52 weeks of monthly dosing, AMG 133 achieved mean weight loss of up to ~20% in adults with obesity (BMI ≥ 30), with no evidence of a plateau by the end of the study period [40]. These findings suggest that AMG 133 may rival the efficacy of leading agents such as retatrutide [36]. By coupling GLP-1 receptor stimulation with GIP receptor blockade, this agent exemplifies a next-generation approach to optimizing incretin-based weight loss therapies. Phase 3 trials, branded as the “MARITIME” programme, are currently in development to further evaluate its long-term efficacy and safety [40].

### 5.3. Amylin Analogues and Amylin Combination Therapies

Amylin is a pancreatic hormone co-secreted with insulin that promotes satiety and slows gastric emptying. Analogues of amylin (acting via amylin/calcitonin receptors) reduce food intake. Alone, amylin agonists cause modest weight loss, but in combination with GLP-1 RAs they may have synergistic effects (as the two hormones together mimic the post-prandial satiety signalling of the gut–pancreas axis) [41].

#### 5.3.1. CagriSema (Cagrilintide + Semaglutide)

CagriSema is a fixed-dose, once-weekly injectable combination of the long-acting amylin analogue cagrilintide and the GLP-1 receptor agonist semaglutide. This dual-hormone approach is designed to harness the complementary mechanisms of amylin and GLP-1 to enhance satiety, reduce food intake, and improve weight loss outcomes. In a 32-week Phase 2 trial involving patients with type 2 diabetes, CagriSema achieved an average weight loss of 15.6%, outperforming both semaglutide 2.4 mg alone (−5.1%) and cagrilintide alone (−8.1%) [42]. More recently, the Phase 3 REDEFINE 1 trial reported even greater efficacy. Under the “efficacy estimand”—which assumes full adherence to treatment—CagriSema led to a mean weight reduction of 22.7% after 68 weeks, compared to 11.8% with cagrilintide 2.4 mg, 16.1% with semaglutide 2.4 mg, and just 2.3% with placebo. Furthermore, 40.4% of participants in the CagriSema group achieved ≥25% weight loss. Using the “treatment policy estimand”—which includes all randomized participants regardless of adherence—CagriSema still demonstrated superior weight loss of 20.4%, versus 11.5% with cagrilintide, 14.9% with semaglutide, and 3.0% with placebo [43]. These findings suggest that CagriSema may offer a significant advancement in obesity pharmacotherapy. If approved, CagriSema could become a new standard for pharmacologic weight management, especially for patients who do not fully respond to GLP-1 RA alone.

#### 5.3.2. Gastrointestinal Peptide-Based Therapies

Beyond GLP-1 and amylin, several other gut-derived peptides are under investigation for their potential roles in obesity treatment. These emerging targets offer novel mechanisms of action that may complement or enhance existing therapies, paving the way for next-generation pharmacologic strategies in weight management.

#### 5.3.3. PYY Analogue (Y14)

Peptide YY_3–36_ (PYY_3–36_), a gut-derived hormone released postprandially from the distal intestine, exerts anorectic effects by binding to hypothalamic Y2 receptors to signal satiety. Y14 is a long-acting PYY_3–36_ analogue designed for extended release. In a Phase 1 clinical trial, overweight participants received up to five weekly subcutaneous injections of Y14. After four weeks, those treated with Y14 lost approximately 2.9–3.6 kg more than the placebo group (*p* < 0.0001). Acute administration of Y14 also reduced food intake by 38–55% compared to placebo. These findings highlight PYY as a promising therapeutic target for appetite suppression and early weight loss [44]. Because the trial lasted only one month, the durability of weight loss with Y14 over longer treatment durations remains unknown, raising questions about the durability of effect over longer treatment durations. As such, PYY analogues may be best suited for combination regimens, potentially augmenting the satiety effects of other agents such as GLP-1 or amylin analogues. To date, no PYY-based therapy has advanced to Phase 3 trials, but the Y14 study provides proof-of-concept that pharmacologic enhancement of endogenous PYY signalling can safely induce meaningful short-term weight loss [44].

#### 5.3.4. ARD-101 (Oral TAS2R Agonist)

ARD-101 is an orally administered formulation of denatonium acetate developed to activate bitter taste receptors (TAS2Rs) in the gastrointestinal tract. In a Phase 1 randomized, double-blind, placebo-controlled trial in healthy adults, ARD-101 was found to be over 99% restricted to the gut, with minimal systemic exposure. The compound was well tolerated at all tested dose levels, and no treatment-related serious adverse events were reported. Blood samples taken one hour after administration of 240 mg ARD-101 showed elevated circulating levels of gut peptide hormones, including GLP-1, peptide YY (PYY), and cholecystokinin (CCK), compared to placebo. These findings support continued clinical evaluation of ARD-101 in metabolic and inflammatory disorders [45].

### 5.4. Other Investigational Therapies

Finally, a growing number of metabolic drugs targeting diverse physiological pathways are currently in development, reflecting the expanding scope of therapeutic strategies beyond traditional gut hormone modulation.

#### 5.4.1. Bimagrumab

Bimagrumab is a monoclonal antibody targeting activin type II receptors, thereby inhibiting signalling by myostatin and related ligands involved in muscle catabolism. Unlike traditional anti-obesity therapies, bimagrumab’s mechanism focuses on improving body composition by increasing skeletal muscle mass while simultaneously reducing fat mass. In a 48-week Phase 2 trial involving adults with obesity and type 2 diabetes, bimagrumab infusions led to a 20.5% reduction in total body fat mass, accompanied by a 3.6% increase in lean mass. This resulted in a net body weight reduction of approximately 6.5% (−5.9 kg), compared to a 0.8% weight gain in the placebo group. Notably, the vast majority of weight lost with bimagrumab was fat—a highly desirable outcome in obesity treatment. The most commonly observed side effects among participants receiving bimagrumab were muscle spasms and mild diarrhea [46]. Although the overall magnitude of weight loss (~6.5%) may be insufficient for bimagrumab to serve as a stand-alone obesity treatment, its unique effect on preserving and increasing lean mass may generate an interest in potential combination strategies. For instance, pairing bimagrumab with a GLP-1 receptor agonist could amplify total weight loss through appetite suppression, while simultaneously mitigating the lean mass loss commonly observed with incretin-based therapies.

Despite the absence of ongoing commercial development for obesity, the therapeutic concept of selectively targeting fat mass while preserving skeletal muscle is gaining recognition [47]. Myostatin inhibition remains a promising avenue for future combination regimens aimed at optimizing both metabolic health and physical resilience during weight loss.

#### 5.4.2. Efinopegdutide (HM15211)

Efinopegdutide, a novel multi-functional therapeutic agent developed by Hanmi Pharmaceutical, is a synthetic peptide of oxyntomodulin conjugated to the Fc region of human IgG4, acting as a dual GLP-1 and glucagon receptor agonist. Designed to target both obesity and nonalcoholic steatohepatitis (NASH), efinopegdutide offers a mechanistically integrated approach to metabolic disease. In a 24-week head-to-head trial involving patients with nonalcoholic fatty liver disease (NAFLD), once-weekly efinopegdutide (titrated to 10 mg) achieved a ~73% reduction in liver fat content, significantly outperforming semaglutide 1 mg, which produced a 42% reduction [48]. While both agents induced substantial weight loss by week 24, the difference was not statistically significant: −8.5% with efinopegdutide versus −7.1% with semaglutide. These results suggest that efinopegdutide achieves comparable weight reduction to semaglutide over six months, with the added benefit of more pronounced effects on hepatic fat metabolism, likely attributable to its FGF21 agonist component. FGF21 analogues are known to enhance energy expenditure, improve insulin sensitivity, and exert hepatoprotective effects [48,49]. Thus, efinopegdutide represents a promising candidate for the dual treatment of obesity and liver-related metabolic dysfunction.

In summary, the pharmacotherapy pipeline for obesity is undergoing a transformative expansion. GLP-1 receptor agonists have become a cornerstone of treatment, and ongoing innovations—such as oral formulations and high-potency analogues—are continually enhancing their therapeutic impact. Building on this foundation, multi-agonist strategies that combine GLP-1 with additional targets such as GIP, glucagon, and amylin are achieving unprecedented levels of weight loss, with some agents inducing reductions of 20–25%, approaching outcomes previously attainable only through bariatric surgery. Meanwhile, emerging therapies that act on entirely different physiological axes—including PYY, FGF21, myostatin, and bitter taste receptors (TAS2Rs)—offer promising adjunctive or alternative approaches. These novel agents may serve to individualize treatment by addressing specific patient needs, such as preserving lean mass, improving hepatic metabolism in NASH, or minimizing gastrointestinal side effects.

As these therapies progress through advanced stages of clinical development, it is evident that the pharmacologic management of obesity is becoming not only more effective but also increasingly sophisticated. This evolving landscape raises critical considerations regarding the role of pharmacotherapy relative to metabolic surgery, including how emerging agents may complement, delay, or in some cases, replace surgical intervention.

Table 1 provides a comprehensive summary of current and emerging anti-obesity pharmacotherapies, detailing their clinical indications, weight loss efficacy, developmental stage, dosing regimens, and mechanisms of action.

Table 2 summarizes the key differences between bariatric surgeries and selected anti-obesity medications, including their efficacy, comorbidity impact, onset of effect, safety profiles, and patient eligibility criteria.

## 6. Future Recommendations

### 6.1. Toward Phenotype-Guided Pharmacotherapy in Obesity Management

Given the rapidly expanding landscape of effective anti-obesity pharmacotherapies, there is a growing need to transition from generalized treatment algorithms toward a phenotype-guided approach to obesity management. Rather than basing treatment decisions solely on BMI thresholds, clinicians should tailor therapy to the predominant clinical phenotype and its associated comorbidities. For example, patients with metabolic dysfunction-associated steatohepatitis (MASH) may benefit from agents such as tirzepatide, mazdutide, or efinopegdutide, all of which have demonstrated significant reductions in hepatic fat and, in some cases, histologic resolution of MASH. Similarly, in patients with obstructive sleep apnea (OSA), tirzepatide has shown the ability to significantly reduce the apnea–hypopnea index and improve sleep-related outcomes. In individuals with cardiovascular disease, both semaglutide and tirzepatide have been shown to reduce major adverse cardiovascular events, while retatrutide and CagriSema may further improve cardiometabolic risk factors. For those with chronic kidney disease (CKD), semaglutide has demonstrated renoprotective effects, including slower decline in eGFR and reduced albuminuria. Patients with osteoarthritis may benefit from the anti-inflammatory and symptom-relieving effects of semaglutide, whereas individuals with sarcopenic obesity—a frequently overlooked phenotype—could be candidates for bimagrumab, which uniquely reduces fat mass while preserving or increasing lean muscle. These examples illustrate how treatment decisions can be optimized by aligning drug mechanisms with the patient’s specific comorbidity profile. Looking ahead, as more agents from diverse pharmacologic classes become available, the development of rational combination therapies is anticipated—particularly for patients in whom a single-agent strategy fails to achieve sufficient weight loss or address the full spectrum of comorbidities.

**Figure 1:**
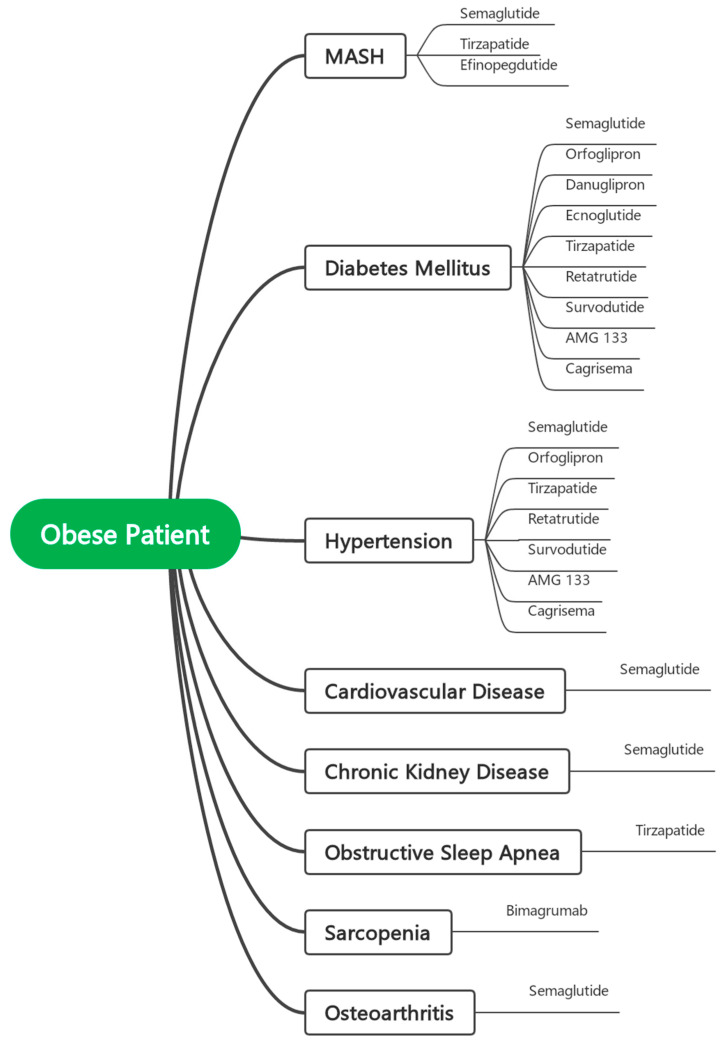
Conceptual Model for Phenotype-Guided Pharmacotherapy in Obesity Management.

Figure 1 presents a conceptual model illustrating the future framework of phenotype-guided pharmacotherapy in obesity management. This figure serves as a visual representation of how treatment selection may be tailored to predominant clinical phenotypes and associated comorbidities. As more anti-obesity agents are approved and integrated into practice, it is anticipated that additional therapeutic links between specific drug classes and diverse obesity-related conditions will emerge, further refining individualized treatment strategies.

### 6.2. Reconsidering Bariatric Surgery Eligibility

Given the rapid evolution of pharmacologic therapies for obesity, it is time to re-evaluate current bariatric surgery eligibility criteria, particularly the BMI thresholds established in the 2022 ASMBS/IFSO guidelines [11]. Under these guidelines, individuals with a BMI ≥ 35 kg/m^2^ (≥32.5 kg/m^2^ in Asian populations) are eligible for bariatric surgery regardless of comorbidities, and those with BMI 30–34.9 kg/m^2^ (27.5–32.4 kg/m^2^ in Asians) may be considered candidates if comorbidities are present. However, the rationale behind lowering these thresholds was largely predicated on the historical absence of effective pharmacologic alternatives—a rationale that is increasingly outdated. With the emergence of potent agents such as semaglutide, tirzepatide, retatrutide, and CagriSema, which can induce substantial and sustained weight loss alongside improvements in metabolic health, the justification for early surgical intervention based solely on BMI is rapidly weakening and will likely continue to erode as more therapies reach clinical use.

Moreover, BMI, while useful as a screening tool, is a limited and often imprecise predictor of long-term outcomes after bariatric surgery. A growing body of evidence supports the use of more comprehensive assessment tools—such as the Edmonton Obesity Staging System (EOSS)—which incorporate comorbidity severity, functional impairment, and psychological status. EOSS has demonstrated greater predictive validity for mortality and surgical outcomes than BMI or waist circumference alone. For instance, patients with EOSS stage 0–1 may have no increased mortality risk despite elevated BMI, whereas those in stage 2–3 carry significantly higher all-cause and cardiovascular mortality [66]. Future guidelines would benefit from incorporating such multidimensional tools to ensure that patients most likely to benefit from surgery are prioritized appropriately.

In this context, we propose that eligibility for bariatric surgery should no longer rely on BMI as the primary eligibility criterion. Rather than reverting to the original pre-2022, more conservative thresholds (e.g., BMI ≥ 40 kg/m^2^ or ≥35 with comorbidities), we recommend a refined, evidence-based framework that raises the BMI cutoff (if not removed and replaced by other tools) while also requiring a mandatory trial of at least one or more phenotype-guided pharmacologic agents prior to surgical consideration—particularly for individuals with BMI between 30 and 40 kg/m^2^. In this model, the selection of pharmacotherapy should be tailored to the patient’s predominant clinical phenotype, such as using tirzepatide for patients with MASH or OSA, or bimagrumab in cases of sarcopenic obesity, as detailed in Figure 1. Surgery should be reserved for those who demonstrate an inadequate response or intolerance to such targeted medical interventions. Exceptions may include patients with severe obesity (e.g., BMI > 40 kg/m^2^) or those requiring urgent and significant weight loss for specific clinical indications, for whom immediate surgical intervention remains appropriate. This approach promotes a rational, stepwise, and individualized model of care, aligning with contemporary understanding of obesity as a complex, chronic, and heterogeneous disease that demands tailored, multimodal treatment strategies.

In conclusion, while current bariatric surgery guidelines consider several clinical parameters, BMI remains the primary determinant of eligibility. However, growing evidence suggests that reliance on BMI as the central criterion may no longer be sufficient to capture the full complexity of obesity and its impact on health. The emergence of highly effective, phenotype-guided anti-obesity pharmacotherapies—capable of producing substantial weight loss and improving a broad spectrum of comorbid conditions—further challenges the appropriateness of proceeding directly to surgery based primarily on anthropometric thresholds. Adopting a more nuanced, individualized framework that prioritizes metabolic health, clinical phenotype, and prior response to pharmacologic agents has the potential to improve patient outcomes and resource allocation. Future guidelines would benefit from integrating multidimensional tools such as the Edmonton Obesity Staging System (EOSS) and requiring targeted medical therapy trials prior to surgical referral—ensuring that bariatric surgery is offered to those most likely to benefit, and that treatment decisions align with the evolving landscape of obesity care.

Table 3 outlines our proposed framework for modernizing bariatric surgery eligibility, shifting from rigid BMI-based thresholds to a more nuanced, patient-centred model anchored in pharmacologic responsiveness and clinical phenotype.

### 6.3. Potential Combination of Pharmacotherapy and Bariatric Surgery

Pharmacotherapy plays an increasingly pivotal role in optimizing outcomes both before and after bariatric surgery. Preoperatively, the use of anti-obesity medications—particularly GLP-1 receptor agonists—can facilitate weight loss prior to surgery. Postoperatively, while bariatric procedures are highly effective, some patients experience suboptimal weight loss or weight regain over time, especially following restrictive procedures such as sleeve gastrectomy. In these cases, pharmacotherapy offers a valuable, non-surgical option to sustain or augment weight loss. Agents such as liraglutide and semaglutide have demonstrated both efficacy and safety in post-bariatric populations, and their use may reduce the need for revision surgery [67]. As evidence grows, integrating pharmacologic strategies into the perioperative care pathway may become standard practice to support long-term success.

## 7. Conclusions

The management of obesity is entering a transformative era. Once dominated by lifestyle modification and limited by surgical accessibility, the therapeutic landscape has expanded dramatically with the emergence of highly effective, phenotype-guided pharmacotherapies. Currently available agents and others in development are now delivering weight loss and comorbidity improvements once thought achievable only through surgery. This evolution demands a fundamental reassessment of bariatric surgery eligibility criteria.

Current guidelines—rooted primarily in BMI thresholds—fail to capture the complexity and heterogeneity of obesity as a chronic, multisystem disease. BMI, while useful for population-level risk stratification, is an imprecise tool for individualized clinical decision-making. More sophisticated approaches such as the Edmonton Obesity Staging System (EOSS) offer a multidimensional view of disease severity and better predict surgical outcomes and mortality risk. In parallel, the growing arsenal of pharmacotherapies allows clinicians to tailor treatment based on a patient’s metabolic phenotype, comorbidity burden, and therapeutic response.

We propose a new framework—outlined in Table 1—that redefines surgical candidacy through three critical shifts: (1) repositioning BMI as a secondary, not primary, determinant of eligibility; (2) requiring a mandatory trial of phenotype-appropriate pharmacotherapy before surgical referral, particularly in patients with BMI 30–40 kg/m^2^; and (3) integrating validated staging tools such as EOSS to guide prioritization. This strategy does not diminish the value of bariatric surgery—it enhances it. By reserving surgical intervention for those with the greatest clinical need and least responsiveness to medical therapy, we elevate both the precision and equity of care.

Ultimately, the future of obesity treatment lies in dismantling outdated binary choices between “medical” and “surgical” pathways. What we need instead is a flexible, layered model of care that adapts to patient-specific trajectories, empowers shared decision-making, and uses every available tool—pharmacologic, behavioural, and surgical—in synergistic alignment. Now is the time to move beyond BMI and toward a new standard that reflects the true biology of obesity and the real potential of modern therapeutics.

## Figures and Tables

**Table 1:** Summary of current and emerging anti-obesity pharmacotherapies.

Drug Name	Potential Clinical Role	Weight Loss Efficacy	Phase/Approval	Route, Dose, Frequency	Mechanism of Action
Semaglutide	Improves glycemic control [16] Lowers blood pressure and lipid levels [50] Lowers kidney disease risk, slows eGFR decline, improves albuminuria, reduces CV events and mortality [16] Improves osteoarthritic pain and function [23] Reduces liver fat content [48] Reduces all-cause mortality [16]	~15% by week 68 [19]	FDA approved [51]	Subcutaneous, 2.4 mg, once weekly [19]	GLP-1 receptor agonist
Orforglipron	Reduces HbA1c level [52] Reduces blood pressure and circulating lipid levels [25]	14.7% by week 36 [25]	Phase 3 trial [25]	Oral tablet, 12–45 mg, once daily [25]	Partial GLP-1 receptor agonist [25]
Danuglipron	Reduces HbA1c level [26]	13% by week 32 [27]	Phase 2 trial [26]	Oral tablet, 200 mg, twice daily [26]	Small-molecule GLP-1 receptor agonist [26]
Ecnoglutide	Reduces HbA1c [29]	13% by week 40 [53]	Phase 3 trial [53]	Subcutaneous, 1.2–2.4 mg, once weekly [53]	Long-acting GLP-1 receptor agonist [29]
Mazdutide	Reduces HbA1c and fasting plasma glucose, lowers blood pressure, total cholesterol, LDL and triglycerides [54]	~14% by week 48 [55]	Phase 3 trial [55]	Subcutaneous, 4–6 mg, once weekly	Dual GLP-1 and GIP receptor agonist [30]
Tirzepatide	Reduces blood glucose levels [33] Reduces systolic blood pressure, circulating lipid levels, and waist circumference [33,34] Reduces Apnea-Hypopnea Index and improves sleep quality in OSA [34] Associated with higher rates of MASH resolution [35]	~21% by week 72 [33]	Phase 3 trial [33]	Subcutaneous, 5–15 mg, once weekly [33]	Dual GLP-1 and GIP receptor agonist [33]
Retatrutide	Reduces HbA1c levels; lowers systolic and diastolic blood pressure; lowers circulating lipid levels [36]	~24% by week 48 [36]	Phase 2 trial [36]	Subcutaneous, 4–12 mg, once weekly [36]	Triple GLP-1, GIP, and glucagon receptor agonist [36]
Survodutide	Improves glucose tolerance and reduces HbA1c levels, while also lowering blood pressure and waist circumference [37].	~15% by week 46 [37]	Phase 3 trial [37]	Subcutaneous, 0.6–4.8 mg, once weekly [37]	Dual GLP-1 and glucagon receptor agonist [37]
VK2735	-	14.7% by week 13 [38]	Phase 3 trial [56]	Subcutaneous, 2.5–15 mg, once weekly [38]	Dual GLP-1 and GIP receptor agonist [38]
VK2735 (oral)	-	8.2% by 28 days [57]	Phase 2 trial [57]	Oral tablet, 100 mg, once daily [57]	Dual GLP-1 and GIP receptor agonist
AMG 133 (Maridebart cafraglutide)	Reduces HbA1c levels, blood pressure, and circulating lipid levels [40].	~20% by week 52 [40]	Phase 3 trial [40]	Subcutaneous, 140–420 mg, once monthly [40]	GLP-1 receptor agonist and GIP receptor antagonist [39]
CagriSema	Reduces HbA1c levels, lowers blood pressure, and improves lipid profile [58].	22.7% by week 68 [43]	Phase 3 trial [43]	Subcutaneous, 2.4 mg of each, once weekly [58]	Amylin analogue + GLP-1 receptor agonist [58].
PYY Analogue (Y14)	-	2.9–3.6 kg more than placebo (4 weeks) [44]	Phase 1 trial [44]	Subcutaneous, 36 mg, 5x weekly [44]	Long-acting PYY3-36 analogue [44]
ARD-101	-	–	Phase 2 trial [45]	Oral tablet, 40–240 mg, once daily [45]	TAS2R agonist [45]
Bimagrumab	Increases skeletal muscle mass [46]	6.5% by week 48 [46]	Phase 2 trial [46]	IV, 10 mg/kg monthly [46]	Activin type II receptor monoclonal antibody [46]
Efinopegdutide	Reduces liver fat content [48]	8.5% by week 24 [48]	Phase 2 trial [48]	Subcutaneous, 10 mg, once weekly [48]	Dual GLP-1 and glucagon receptor agonist [48]

**Table 2:** Summary of bariatric surgeries and selected anti-obesity pharmacotherapies, comparing weight loss efficacy, comorbidity impact, time to response, side effects, reversibility, eligibility criteria, and other clinical considerations.

	Roux-en-Y Gastric Bypass	Sleeve Gastrectomy	GLP-1 RA (Semaglutide)	Dual Agonist (Tirzepatide)	Triple Agonist (Retatrutide)
Total Weight Loss%	Up to ~31% [59,60]	Up to ~22% [61,62]	~15% [19]	~21% [33]	~24.2% (weight loss continued without plateauing at the end of the trial) [36]
Impact on Comorbidities	High remission rate of T2DM, HTN, OSA [63]	High remission rate of T2DM, HTN, OSA [63]	Excellent effect on glycemic control; Reduction in cardiovascular risk, Slows the progression of Chronic Kidney Disease [20,21,22]	Significant improvement in Obstructive Sleep Apnea, Blood pressure, MASH, Glycemic [33,34,35]	Significant Improvement of Glycemic markers, blood pressure, lipid profile, and quality of life [36]
Time to Response	rapid during the first 6 to 12 months; the greatest weight reduction typically occurred within the first year [64]	Rapid, but slightly slower than Roux-en-Y [64]	Weight loss began by week 4, Reached its lowest point (maximum loss) at week 60 [19]	Rapid and substantial weight loss; exact onset not specified [33]	Rapid drop at 48 weeks; plateau not yet reached [36]
Side Effects/Complications	Leaks, bleeding, bowel obstruction, deep vein thrombosis, pulmonary embolism, nutritional deficiencies [65]	abscess, bleeding, stricture, splenic injury, choledocholithiasis and bile duct stricture [65]	GI: Nausea and diarrhea—typically transient and mild-to-moderate in severity and subsided with time [19].	mild-to-moderate GI symptoms (nausea, diarrhea, constipation) during dose escalation; rare AEs included cholecystitis (≤0.6%) and non-severe pancreatitis [33]	GI: nausea, diarrhea, constipation (generally mild/moderate during dose escalation) [36] Cutaneous hyperesthesia (7% of the patients) [36]
Reversibility	No	No	Yes	Yes	Yes
Eligibility Criteria	BMI ≥ 35 or ≥30 w/comorbidity [11]	BMI ≥ 35 or ≥30 w/comorbidity [11]	BMI ≥ 30 or ≥27 w/comorbidity [51]	Same as GLP-1 RA [33]	Trial-only (FDA approval pending) [36]
Mode of Administration	Invasive surgery	Invasive surgery	Weekly injection [19]	Weekly injection [33]	Weekly injection [36]

**Table 3:** Reimagining Bariatric Surgery Eligibility in the Era of Advanced Pharmacotherapy.

Component	Current Standard (ASMBS/IFSO 2022 [11])	Proposed Framework	Clinical Example
Primary Determinant of Eligibility	BMI-centric (BMI ≥ 35 kg/m^2^ regardless of comorbidities; ≥30 kg/m^2^ with comorbidities)	Multidimensional: BMI considered alongside metabolic health, functional status, treatment history, and comorbidity burden	Patient with BMI 33 and EOSS Stage 0 may not qualify; patient with BMI 32 and EOSS Stage 3 should be prioritized
Pharmacotherapy Requirement	Not required before surgery	Mandatory trial of ≥1 phenotype-guided agent prior to surgical referral for BMI 30–40 kg/m^2^	Patient with BMI 37 and osteoarthritis trials semaglutide; surgery only if functional status or weight trajectory remains suboptimal
BMI ≥ 35 kg/m^2^	Eligible for surgery regardless of comorbidities	Eligible after documented pharmacotherapy trial and phenotype-based assessment	Patient with BMI 36 and MASH treated with tirzepatide or efinopegdutide prior to surgical consideration
BMI 30–34.9 kg/m^2^	Eligible if comorbidities are present	Eligible only after unsuccessful phenotype-guided pharmacotherapy and confirmed metabolic or functional impairment	Patient with BMI 33 and OSA initiates tirzepatide; surgery considered only if no substantial improvement in AHI after 6–12 months
BMI > 40 kg/m^2^	Eligible regardless of clinical context	Still eligible for immediate surgery, but pharmacotherapy encouraged as adjunct where appropriate	Patient with BMI 44 and uncontrolled diabetes may proceed directly to surgery if pharmacologic delay poses risk
Phenotype Integration	Not formally integrated	Drug selection and timing individualized based on obesity phenotype (e.g., MASH, sarcopenia, CKD, OSA)	Bimagrumab for sarcopenic obesity; tirzepatide for MASH/OSA; efinopegdutide for NASH; retatrutide for severe cardiometabolic risk
Assessment Tools	BMI ± Comorbidity status	Add Edmonton Obesity Staging System (EOSS) or equivalent to better stratify surgical risk and prioritize patient benefit	EOSS Stage 2–3 patient may warrant surgery despite lower BMI, while EOSS Stage 0–1 may benefit from continued non-invasive management
Exceptions to Pharmacotherapy Trial	Not explicitly outlined	Immediate surgery permitted in specific scenarios requiring urgent intervention or where medications are contraindicated	Examples: Transplant candidates, patients with severe OSA needing rapid decompression, or those intolerant to GLP-1 RAs or other agents

## Data Availability

This review did not generate any new data. All data referenced are from published studies and publicly available sources, as cited within the article.

## References

[1] Boutari C., Mantzoros C.S. (2022). A 2022 update on the epidemiology of obesity and a call to action: As its twin COVID-19 pandemic appears to be receding, the obesity and dysmetabolism pandemic continues to rage on. Metabolism.

[2] Masood B., Moorthy M. (2023). Causes of obesity: A review. Clin. Med..

[3] WHO (2025). Obesity and Overweight.

[4] Prospective Studies Collaboration (2009). Body-mass index and cause-specific mortality in 900 000 adults: Collaborative analyses of 57 prospective studies. Lancet.

[5] Rodgers R.J., Tschöp M.H., Wilding J.P.H. (2012). Anti-obesity drugs: Past, present and future. Dis. Model. Mech..

[6] Coutinho W., Halpern B. (2024). Pharmacotherapy for obesity: Moving towards efficacy improvement. Diabetol. Metab. Syndr..

[7] Angrisani L., Santonicola A., Iovino P., Formisano G., Buchwald H., Scopinaro N. (2015). Bariatric Surgery Worldwide 2013. Obes. Surg..

[8] Aljulifi M.Z. (2021). Prevalence and reasons of increased type 2 diabetes in Gulf Cooperation Council Countries. Saudi Med. J..

[9] Patti M.E. (2023). Triple G Agonists—A Home Run for Obesity?. N. Engl. J. Med..

[10] Hubbard V.S., Hall W.H. (1991). Gastrointestinal Surgery for Severe Obesity 25–27 March 1991. Obes. Surg..

[11] Eisenberg D., Shikora S.A., Aarts E., Aminian A., Angrisani L., Cohen R.V., De Luca M., Faria S.L., Goodpaster K.P.S., Haddad A. (2023). 2022 American Society of Metabolic and Bariatric Surgery (ASMBS) and International Federation for the Surgery of Obesity and Metabolic Disorders (IFSO) Indications for Metabolic and Bariatric Surgery. Obes. Surg..

[12] Lloyd S.J.-A., Wall-Wieler E., Liu Y., Zheng F., LaMasters T. (2025). Unveiling the cost-effectiveness of bariatric surgery: Insights from a matched cohort study. Surgery for Obesity and Related Diseases.

[13] Ju T., Rivas L., Arnott S., Olafson S., Whitlock A., Sparks A., Haskins I.N., Lin P.P., Vaziri K. (2019). Barriers to bariatric surgery: Factors influencing progression to bariatric surgery in a U.S. metropolitan area. Surg. Obes. Relat. Dis..

[14] Hlavin C., Sebastiani R.S., Scherer R.J., Kenkre T., Bernardi K., Reed D.A., Ahmed B., Courcoulas A. (2023). Barriers to Bariatric Surgery: A Mixed Methods Study Investigating Obstacles Between Clinic Contact and Surgery. Obes. Surg..

[15] Aronne L.J., Horn D.B., Le Roux C.W., Ho W., Falcon B.L., Gomez Valderas E., Das S., Lee C.J., Glass L.C., Senyucel C. (2025). Tirzepatide as Compared with Semaglutide for the Treatment of Obesity. N. Engl. J. Med..

[16] Perkovic V., Tuttle K.R., Rossing P., Mahaffey K.W., Mann J.F.E., Bakris G., Baeres F.M.M., Idorn T., Bosch-Traberg H., Lausvig N.L. (2024). Effects of Semaglutide on Chronic Kidney Disease in Patients with Type 2 Diabetes. N. Engl. J. Med..

[17] Jensterle M., Rizzo M., Haluzík M., Janež A. (2022). Efficacy of GLP-1 RA Approved for Weight Management in Patients with or Without Diabetes: A Narrative Review. Adv. Ther..

[18] Trujillo J.M., Nuffer W., Smith B.A. (2021). GLP-1 receptor agonists: An updated review of head-to-head clinical studies. Ther. Adv. Endocrinol. Metab..

[19] Wilding J.P.H., Batterham R.L., Calanna S., Davies M., Van Gaal L.F., Lingvay I., McGowan B.M., Rosenstock J., Tran M.T.D., Wadden T.A. (2021). Once-Weekly Semaglutide in Adults with Overweight or Obesity. N. Engl. J. Med..

[20] Husain M., Birkenfeld A.L., Donsmark M., Dungan K., Eliaschewitz F.G., Franco D.R., Jeppesen O.K., Lingvay I., Mosenzon O., Pedersen S.D. (2019). Oral Semaglutide and Cardiovascular Outcomes in Patients with Type 2 Diabetes. N. Engl. J. Med..

[21] Marso S.P., Bain S.C., Consoli A., Eliaschewitz F.G., Jódar E., Leiter L.A., Lingvay I., Rosenstock J., Seufert J., Warren M.L. (2016). Semaglutide and Cardiovascular Outcomes in Patients with Type 2 Diabetes. N. Engl. J. Med..

[22] McGuire D.K., Marx N., Mulvagh S.L., Deanfield J.E., Inzucchi S.E., Pop-Busui R., Mann J.F.E., Emerson S.S., Poulter N.R., Engelmann M.D.M. (2025). Oral Semaglutide and Cardiovascular Outcomes in High-Risk Type 2 Diabetes. N. Engl. J. Med..

[23] Bliddal H., Bays H., Czernichow S., Uddén Hemmingsson J., Hjelmesæth J., Hoffmann Morville T., Koroleva A., Skov Neergaard J., Vélez Sánchez P., Wharton S. (2024). Once-Weekly Semaglutide in Persons with Obesity and Knee Osteoarthritis. N. Engl. J. Med..

[24] Medhati P., Shin T.H., Wasden K., Mathur V., Apovian C., Nimeri A., Sheu E.G., Tavakkoli A. (2025). GLP-1RA in the Real World: 1-year Compliance and Outcomes of Semaglutide use in Patients With or Without Previous History of Bariatric Surgery. Ann. Surg..

[25] Wharton S., Blevins T., Connery L., Rosenstock J., Raha S., Liu R., Ma X., Mather K.J., Haupt A., Robins D. (2023). Daily Oral GLP-1 Receptor Agonist Orforglipron for Adults with Obesity. N. Engl. J. Med..

[26] Saxena A.R., Frias J.P., Gorman D.N., Lopez R.N., Andrawis N., Tsamandouras N., Birnbaum M.J. (2023). Tolerability, safety and pharmacodynamics of oral, small-molecule glucagon-like peptide-1 receptor agonist danuglipron for type 2 diabetes: A 12-week, randomized, placebo-controlled, Phase 2 study comparing different dose-escalation schemes. Diabetes Obes. Metab..

[27] Pfizer (2023). Pfizer Announces Topline Phase 2b Results of Oral GLP-1R Agonist, Danuglipron, in Adults with Obesity. Pfizer. https://www.pfizer.com/news/press-release/press-release-detail/pfizer-announces-topline-phase-2b-results-oral-glp-1r#:~:text=Pfizer%20Announces%20Topline%20Phase%202b,at%2026%20weeks.

[28] Pfizer (2024). Pfizer Advances Development of Once-Daily Formulation of Oral GLP-1 Receptor Agonist Danuglipron. Pfizer. https://www.pfizer.com/news/press-release/press-release-detail/pfizer-advances-development-once-daily-formulation-oral-glp.

[29] Zhu D., Wang W., Tong G., Ma G., Ma J., Han J., Zhang X., Liu Y., Gan S., Qin H. (2024). Efficacy and safety of GLP-1 analog ecnoglutide in adults with type 2 diabetes: A randomized, double-blind, placebo-controlled phase 2 trial. Nat. Commun..

[30] Ji L., Jiang H., Cheng Z., Qiu W., Liao L., Zhang Y., Li X., Pang S., Zhang L., Chen L. (2023). A phase 2 randomised controlled trial of mazdutide in Chinese overweight adults or adults with obesity. Nat. Commun..

[31] Jiang H., Ji L., Zhang Y., Cheng Z., Pang S., Li X., Qiu W., Ma Q., Liu Z., Wang Y. (2024). 1866-LB: A Phase 2 Study of Mazdutide 9 mg in Chinese Adults with BMI of 30 kg/m2 or more. Diabetes.

[32] Gutgesell R.M., Nogueiras R., Tschöp M.H., Müller T.D. (2024). Dual and Triple Incretin-Based Co-agonists: Novel Therapeutics for Obesity and Diabetes. Diabetes Ther..

[33] Jastreboff A.M., Aronne L.J., Ahmad N.N., Wharton S., Connery L., Alves B., Kiyosue A., Zhang S., Liu B., Bunck M.C. (2022). Tirzepatide Once Weekly for the Treatment of Obesity. N. Engl. J. Med..

[34] Malhotra A., Grunstein R.R., Fietze I., Weaver T.E., Redline S., Azarbarzin A., Sands S.A., Schwab R.J., Dunn J.P., Chakladar S. (2024). Tirzepatide for the Treatment of Obstructive Sleep Apnea and Obesity. N. Engl. J. Med..

[35] Loomba R., Hartman M.L., Lawitz E.J., Vuppalanchi R., Boursier J., Bugianesi E., Yoneda M., Behling C., Cummings O.W., Tang Y. (2024). Tirzepatide for Metabolic Dysfunction–Associated Steatohepatitis with Liver Fibrosis. N. Engl. J. Med..

[36] Jastreboff A.M., Kaplan L.M., Frías J.P., Wu Q., Du Y., Gurbuz S., Coskun T., Haupt A., Milicevic Z., Hartman M.L. (2023). Triple–Hormone-Receptor Agonist Retatrutide for Obesity—A Phase 2 Trial. N. Engl. J. Med..

[37] Le Roux C.W., Steen O., Lucas K.J., Startseva E., Unseld A., Hennige A.M. (2024). Glucagon and GLP-1 receptor dual agonist survodutide for obesity: A randomised, double-blind, placebo-controlled, dose-finding phase 2 trial. Lancet Diabetes Endocrinol..

[38] Viking (2024). Viking Therapeutics Announces Positive Top-Line Results from Phase 2 VENTURE Trial of Dual GLP-1/GIP Receptor Agonist VK2735 in Patients with Obesity [Internet]. Viking. https://ir.vikingtherapeutics.com/2024-02-27-Viking-Therapeutics-Announces-Positive-Top-Line-Results-from-Phase-2-VENTURE-Trial-of-Dual-GLP-1-GIP-Receptor-Agonist-VK2735-in-Patients-with-Obesity.

[39] Véniant M.M., Lu S.-C., Atangan L., Komorowski R., Stanislaus S., Cheng Y., Wu B., Falsey J.R., Hager T., Thomas V.A. (2024). A GIPR antagonist conjugated to GLP-1 analogues promotes weight loss with improved metabolic parameters in preclinical and phase 1 settings. Nat. Metab..

[40] Agmen (2024). Amgen Announces Robust Weight Loss with MARITIDE in People Living with Obesity or Overweight at 52 Weeks in a Phase 2 Study. AGMEN. https://www.amgen.com/newsroom/press-releases/2024/11/amgen-announces-robust-weight-loss-with-maritide-in-people-living-with-obesity-or-overweight-at-52-weeks-in-a-phase-2-study#:~:text=announced%20positive%20data%20at%2052,also%20without%20a%20weight%20loss.

[41] Dehestani B., Stratford N.R., Roux C.W.L. (2021). Amylin as a Future Obesity Treatment. J. Obes. Metab. Syndr..

[42] Frias J.P., Deenadayalan S., Erichsen L., Knop F.K., Lingvay I., Macura S., Mathieu C., Pedersen S.D., Davies M. (2023). Efficacy and safety of co-administered once-weekly cagrilintide 2·4 mg with once-weekly semaglutide 2·4 mg in type 2 diabetes: A multicentre, randomised, double-blind, active-controlled, phase 2 trial. Lancet.

[43] Novo Nordisk (2024). Novo Nordisk A/S: CagriSema Demonstrates Superior Weight Loss in Adults with Obesity or Overweight in the REDEFINE 1 Trial [Internet]. Novo Nordisk. https://www.novonordisk.com/news-and-media/news-and-ir-materials/news-details.html?id=915082..

[44] Tan T.M.-M., Minnion J., Khoo B., Ball L., Malviya R., Day E., Fiorentino F., Brindley C., Bush J., Bloom S.R. (2021). Safety and efficacy of an extended-release peptide YY analogue for obesity: A randomized, placebo-controlled, phase 1 trial. Diabetes Obes. Metab..

[45] Niethammer A.G., Zheng Z., Timmer A., Lee T. (2022). First-in-Human Evaluation of Oral Denatonium Acetate (ARD-101), a Potential Bitter Taste Receptor Agonist: A Randomized, Double-Blind, Placebo-Controlled Phase 1 Trial in Healthy Adults. Clin. Pharmacol. Drug Dev..

[46] Heymsfield S.B., Coleman L.A., Miller R., Rooks D.S., Laurent D., Petricoul O., Praestgaard J., Swan T., Wade T., Perry R.G. (2021). Effect of Bimagrumab vs Placebo on Body Fat Mass Among Adults with Type 2 Diabetes and Obesity: A Phase 2 Randomized Clinical Trial. JAMA Netw. Open.

[47] Conte C., Hall K.D., Klein S. (2024). Is Weight Loss–Induced Muscle Mass Loss Clinically Relevant?. JAMA.

[48] Romero-Gómez M., Lawitz E., Shankar R.R., Chaudhri E., Liu J., Lam R.L.H., Kaufman K.D., Engel S.S., Bruzone S.O., Coronel M.J. (2023). A phase IIa active-comparator-controlled study to evaluate the efficacy and safety of efinopegdutide in patients with non-alcoholic fatty liver disease. J. Hepatol..

[49] Pan Q., Lin S., Li Y., Liu L., Li X., Gao X., Yan J., Gu B., Chen X., Li W. (2021). A novel GLP-1 and FGF21 dual agonist has therapeutic potential for diabetes and non-alcoholic steatohepatitis. EBioMedicine.

[50] Kosiborod M.N., Bhatta M., Davies M., Deanfield J.E., Garvey W.T., Khalid U., Kushner R., Rubino D.M., Zeuthen N., Verma S. (2023). Semaglutide improves cardiometabolic risk factors in adults with overweight or obesity: STEP 1 and 4 exploratory analyses. Diabetes Obes. Metab..

[51] Chao A.M., Tronieri J.S., Amaro A., Wadden T.A. (2023). Semaglutide for the treatment of obesity. Trends Cardiovasc. Med..

[52] Dutta D., Nagendra L., Anne B., Kumar M., Sharma M., Kamrul-Hasan A.B.M. (2024). Orforglipron, a novel non-peptide oral daily glucagon-like peptide-1 receptor agonist as an anti-obesity medicine: A systematic review and meta-analysis. Obes. Sci. Amp Pract..

[53] Ji L., Gao L., Xue H., Tian J., Wang K., Jiang H., Huang C., Lian Q., Yuan M., Gao G. (2025). Efficacy and safety of a biased GLP-1 receptor agonist ecnoglutide in adults with overweight or obesity: A multicentre, randomised, double-blind, placebo-controlled, phase 3 trial. Lancet Diabetes Endocrinol..

[54] Nalisa D.L., Cuboia N., Dyab E., Jackson I.L., Felix H.J., Shoki P., Mubiana M., Oyedeji-Amusa M., Azevedo L., Jiang H. (2024). Efficacy and safety of Mazdutide on weight loss among diabetic and non-diabetic patients: A systematic review and meta-analysis of randomized controlled trials. Front. Endocrinol..

[55] Ji L., Jiang H., Bi Y., Li H., Tian J., Liu D., Zhao Y., Qiu W., Huang C., Chen L. (2025). Once-Weekly Mazdutide in Chinese Adults with Obesity or Overweight. N. Engl. J. Med..

[56] (2025). Viking Therapeutics Announces Initiation of Phase 3 Obesity Clinical Program with GLP-1/GIP Agonist VK2735. Viking Therapeutics. https://ir.vikingtherapeutics.com/2025-06-25-Viking-Therapeutics-Announces-Initiation-of-Phase-3-Obesity-Clinical-Program-with-GLP-1-GIP-Agonist-VK2735.

[57] (2025). Viking Therapeutics Announces Initiation of Phase 2 VENTURE-Oral Dosing Trial of VK2735 Tablet Formulation in Patients with Obesity. https://ir.vikingtherapeutics.com/2025-01-08-Viking-Therapeutics-Announces-Initiation-of-Phase-2-VENTURE-Oral-Dosing-Trial-of-VK2735-Tablet-Formulation-in-Patients-with-Obesity.

[58] Dutta D., Nagendra L., Harish B., Sharma M., Joshi A., Hathur B., Kamrul-Hasan A. (2024). Efficacy and Safety of Cagrilintide Alone and in Combination with Semaglutide (Cagrisema) as Anti-Obesity Medications: A Systematic Review and Meta-Analysis. Indian J. Endocrinol. Metab..

[59] Chang S.-H., Gasoyan H., Wang M., Ackermann N., Liu X., Herrick C., Eckhouse S., Dimou F., Vuong L., Colditz G.A. (2022). 10-year weight loss outcomes after Roux-en-Y gastric bypass and attendance at follow-up visits: A single-center study. Surg. Obes. Relat. Dis..

[60] Shubeck S., Dimick J.B., Telem D.A. (2018). Long-term Outcomes Following Bariatric Surgery. JAMA.

[61] Musella M., Berardi G., Velotti N., Schiavone V., Vitiello A. (2021). Ten-Year Results of Laparoscopic Sleeve Gastrectomy: Retrospective Matched Comparison with Laparoscopic Adjustable Gastric Banding—Is There a Significant Difference in Long Term?. Obes. Surg..

[62] Dowgiałło-Gornowicz N., Jaworski P., Orłowski M., Franczak P., Proczko-Stepaniak M., Kloczkowska A., Karpińska I., Lech P., Major P. (2025). Long-term outcomes of metabolic bariatric surgery: A 10-Year multicenter retrospective study in Poland (BARI-10-POL). Langenbecks Arch. Surg..

[63] Castanha C.R., Tcbc-Pe Á.A.B.F., Castanha A.R., Belo G.D.Q.M.B., Lacerda R.M.R., Vilar L. (2018). Avaliação da qualidade de vida, perda de peso e comorbidades de pacientes submetidos à cirurgia bariátrica. Rev. Col. Bras. Cir..

[64] Wolfe B.M., Kvach E., Eckel R.H. (2016). Treatment of Obesity: Weight Loss and Bariatric Surgery. Circ. Res..

[65] Noria S., Grantcharov T. (2013). Biological effects of bariatric surgery on obesity-related comorbidities. Can. J. Surg..

[66] Segal-Lieberman G., Segal P., Dicker D. (2016). Revisiting the Role of BMI in the Guidelines for Bariatric Surgery. Diabetes Care.

[67] Horváth L., Mráz M., Jude E.B., Haluzík M. (2024). Pharmacotherapy as an Augmentation to Bariatric Surgery for Obesity. Drugs.

